# O-GlcNAc transferase maintains metabolic homeostasis in response to CDK9 inhibition

**DOI:** 10.1093/glycob/cwac038

**Published:** 2022-06-16

**Authors:** Aishwarya Gondane, Ninu Poulose, Suzanne Walker, Ian G Mills, Harri M Itkonen

**Affiliations:** Department of Biochemistry and Developmental Biology, Faculty of Medicine, University of Helsinki, Helsinki 00014, Finland; Patrick G Johnston Centre for Cancer Research, Queen’s University, Belfast BT9 7AE, United Kingdom; Nuffield Department of Surgical Sciences, John Radcliffe Hospital, University of Oxford, Oxford OX3 9DU, United Kingdom; Department of Microbiology, Blavatnik Institute, Harvard Medical School, Boston, MA 02115, United States; Patrick G Johnston Centre for Cancer Research, Queen’s University, Belfast BT9 7AE, United Kingdom; Nuffield Department of Surgical Sciences, John Radcliffe Hospital, University of Oxford, Oxford OX3 9DU, United Kingdom; Department of Biochemistry and Developmental Biology, Faculty of Medicine, University of Helsinki, Helsinki 00014, Finland

**Keywords:** cyclin-dependent kinase 9, metabolism, O-GlcNAc transferase, prostate cancer, systems biology

## Abstract

Co-targeting of O-GlcNAc transferase (OGT) and the transcriptional kinase cyclin-dependent kinase 9 (CDK9) is toxic to prostate cancer cells. As OGT is an essential glycosyltransferase, identifying an alternative target showing similar effects is of great interest. Here, we used a multiomics approach (transcriptomics, metabolomics, and proteomics) to better understand the mechanistic basis of the combinatorial lethality between OGT and CDK9 inhibition. CDK9 inhibition preferentially affected transcription. In contrast, depletion of OGT activity predominantly remodeled the metabolome. Using an unbiased systems biology approach (weighted gene correlation network analysis), we discovered that CDK9 inhibition alters mitochondrial activity/flux, and high OGT activity is essential to maintain mitochondrial respiration when CDK9 activity is depleted. Our metabolite profiling data revealed that pantothenic acid (vitamin B5) is the metabolite that is most robustly induced by both OGT and OGT+CDK9 inhibitor treatments but not by CDK9 inhibition alone. Finally, supplementing prostate cancer cell lines with vitamin B5 in the presence of CDK9 inhibitor mimics the effects of co-targeting OGT and CDK9.

## 1. Introduction

Prostate cancer is the most common cancer in men. Progression of prostate cancer is driven by the nuclear hormone transcription factor, androgen receptor (AR). AR-mediated metabolic reprogramming promotes proliferation of prostate cancer cells by positively regulating anabolic metabolism and lipogenesis (Barfeld et al. 2014; Bader and McGuire 2020; Ahmad et al. 2021). Depletion of AR activity halts the proliferation of prostate cancer cells. However, in a significant number of cases, resistance to AR-targeted therapies arises; therefore, there is a need to discover new ways to treat prostate cancer.

We have shown that O-GlcNAc transferase (OGT) is overexpressed in prostate cancer, making this enzyme a potential drug target for the disease (Itkonen et al. 2013; Itkonen et al. 2021). *OGT* is an essential gene in the higher eukaryotes, and the enzyme catalyzes all nucleocytoplasmic O-GlcNAcylation of thousands of candidate substrate proteins (Levine and Walker 2016; Yang and Qian 2017; Itkonen et al. 2021). However, in most cases, the context-specific role of OGT is not well understood. Because OGT is an essential gene, knockout of the enzyme kills all cells (Kazemi et al. 2010; Itkonen et al. 2021). This necessitates the development of the specific small molecule inhibitors. OGT small molecule inhibitors 2 and 4 (OSMI-2 and OSMI-4) are currently the most specific small molecule inhibitors targeting OGT (Martin et al. 2018; Itkonen et al. 2021).

Compounds targeting OGT have minimal effects on the proliferation rate of prostate cancer cells but have profound effects on the mitochondrial activity of these cells (Itkonen et al. 2016; Itkonen et al. 2020). This implies that prostate cancer cells mount an adaptive response that enables their survival despite the diminished OGT activity. To understand this adaptive response, we performed a combinatorial lethality screen. The screen revealed a novel way to selectively kill prostate cancer cells by combining OGT inhibitor with compounds targeting the transcription elongation kinase, cyclin-dependent kinase 9 (CDK9; Itkonen et al. 2020).

CDK9 phosphorylates the C-terminal domain of RNA polymerase II (RNA Pol II) to promote productive transcription elongation (Chou et al. 2020). CDK9 and OGT both have roles in transcription, and combinatorial inhibition of the two further suppresses RNA Pol II activity (Itkonen et al. 2020; Hu et al. 2021). Interestingly, combinatorial targeting of OGT and CDK9 is selectively toxic to prostate cancer cells but not to normal prostate cells (Itkonen et al. 2020). Since all cells depend on transcription, effects on transcription cannot fully explain the combinatorial lethal effects. The central dogma states that replication, transcription, and translation are the three major processes that sustain biological information and ultimately form the framework to understand why certain compounds halt the proliferation of cells.

In this study, we have employed a multiomics approach to understand how prostate cancer cells rewire transcriptional, proteomic, and metabolic networks when CDK9 and OGT are inhibited. Using an unbiased systems biology approach, weighted gene correlation network analysis (WGCNA), we discovered that CDK9 inhibitor-induced metabolic remodeling is dependent on OGT. Our study underscores the importance of multiomics approaches to understand how cells respond to a perturbation.

## 2. Results

### 2.1. Effects of OGT and CDK9 inhibition on the metabolome, transcriptome, and proteome

**Fig. 1:**
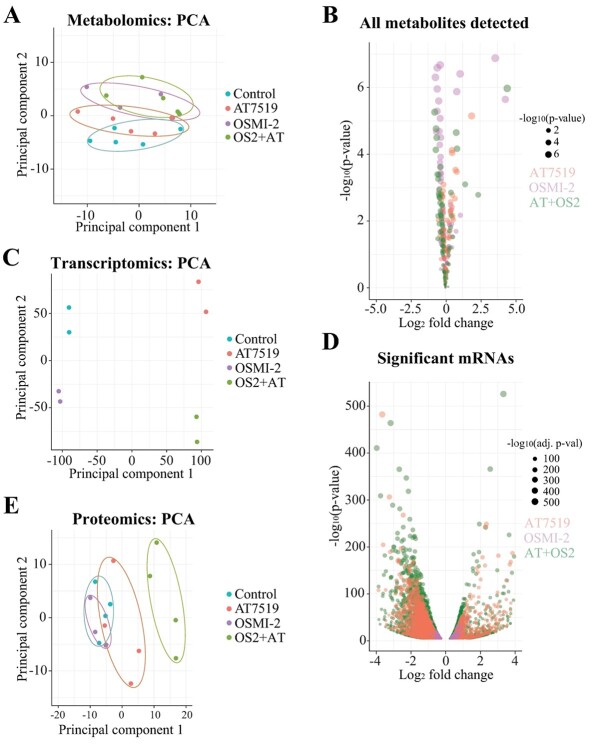
OGT inhibition affects metabolic processes in prostate cancer cells. PCA is presented in 1A, 1C, and 1E, while 1B shows all the metabolites detected, and 1D shows the significantly affected mRNAs. A) LNCaP cells were treated for 24 h with inhibitors of CDK9 (0.5 μM AT7519), OGT (40 μM OSMI-2), and CDK9 + OGT followed by metabolite profiling. Clustering of the metabolomics data using PCA. B) Volcano plot showing the fold change in metabolite levels in the AT7519 (orange), OSMI-2 (lilac), and the combination (green) treatments. The size of the circles depicts the *P*-values. C) PCA of the transcriptome profiles of CDK9, OGT, and CDK9 + OGT inhibitor-treated samples (doses as in A). RNA-seq data were downloaded from GSE116778. D) Volcano plot of fold changes in gene expression in the AT7519 (orange), OSMI-2 (lilac), and combination (green) treatments. The size of the circles depicts the *P*-values. E) LNCaP cells were treated as in A and analyzed using reverse-phase protein arrays. Shown is the PCA.

We have shown that combined inhibition of OGT and CDK9 is toxic to prostate cancer cells (Itkonen et al. 2020), and here we used a multiomics approach to explain the mechanistic basis for the combinatorial effects. The combinatorial lethality was observed in prostate cancer cells treated with an OGT inhibitor (OSMI-2) (Martin et al. 2018) and a CDK9 inhibitor (AT7519) (Wyatt et al. 2008). First, we performed metabolite profiling of LNCaP prostate cancer cells after 24-h treatment with these compounds. Principal component analysis (PCA) showed that OGT and the OGT + CDK9 inhibitor-treated samples are clustered clearly distinct from the controls (Fig. 1A). In contrast, the CDK9 inhibitor-treated samples and the control samples did not separate clearly. These data show that the OGT inhibition exerts a more significant effect on metabolite levels than CDK9 inhibition. In general, OGT inhibition decreased the abundance of most metabolites (Fig. 1B). By co-targeting OGT and CDK9, we noted that the general metabolite levels were reduced even more significantly than for the single-agent treatments (Fig. 1B and Supplementary Fig. 1A).

To assess the transcriptional effects of targeting OGT and CDK9, we used a previously published RNA-seq dataset (Itkonen et al. 2020) generated from the same cell line with the same compounds as we used for metabolite profiling. PCA showed that the OGT inhibitor-treated samples cluster with the control samples (Fig. 1C). In contrast, CDK9 inhibitor-treated samples were distinct from any of the other samples. Cells that were co-treated with OGT and CDK9 inhibitors formed a distinct cluster. This implies that the contribution of OGT to the regulation of transcription becomes more significant when cells are experiencing transcriptional stress.

More detailed analysis of the RNA-seq data showed that the OGT inhibitor has minimal effects on the transcriptome as a single agent, affecting the expression of a relatively small number of mRNAs (Fig. 1D and Supplementary Fig. 1B). However, and as previously reported (Park et al. 2017), OGT mRNA itself was induced by OGT inhibition (Supplementary Fig. 2). As expected, CDK9 inhibition had robust effects on the transcriptome of the cell, with most mRNAs downregulated (Fig. 1D). Finally, combining OGT and CDK9 inhibitors further downregulated transcription, as previously reported (Itkonen et al. 2020).

**Fig. 2:**
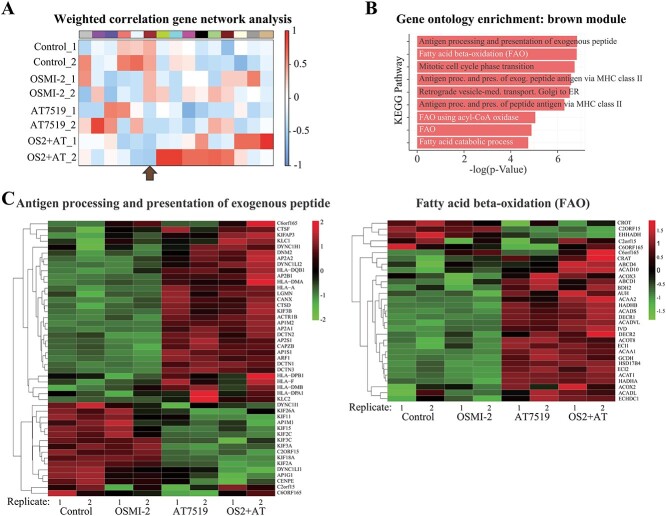
Systems biology approach to identify critical processes in response to CDK9 inhibition. A) Module trait relationship heatmap derived from WGCNA of the transcriptomics data presented in Fig. 1A. Genes clustered in the brown module positively correlate to the control and the OGT inhibitor-treated conditions. In contrast, they show negative correlation with the CDK9 inhibitor and combination treatments. B) Gene ontology enrichment analysis of the genes clustered in the brown module. The “antigen processing and presentation of exogenous peptides” and “fatty acid beta oxidation” are the most significantly enriched processes. C) Gene expression heatmaps of the genes enriched for the “antigen processing and presentation of exogenous peptides” and “fatty acid beta oxidation.”

We used reverse-phase protein arrays to profile the proteomic response to CDK9 inhibition. Combining OGT and CDK9 inhibitors had more widespread effects than either of the single-agent treatments as determined using the PCA (Fig. 1E). In addition, the combination further enhanced the effects on the proteins known to be affected by OGT inhibition, including cyclin-dependent kinase 1, cyclin B1, and polo-like kinase 1 (Itkonen, Urbanucci, et al. 2019b), and CDK9 inhibition (phosphorylation of RB1; Squires et al. 2009, Supplementary Fig. 3A). Interestingly, we noted that only the co-treatment induced significant upregulation of the immune signaling transcription factor, interferon regulatory factor 1 (IRF1; Supplementary Fig. 3B).

**Fig. 3:**
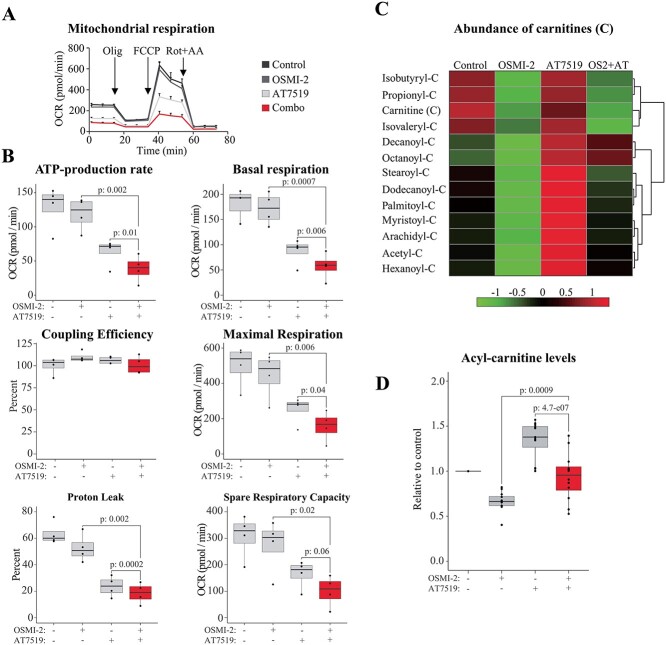
Co-targeting of OGT and CDK9 reduces mitochondrial activity. A, B) Oxygen consumption rate (OCR) was measured using Seahorse XFe 96 instrument. The OCR is used to estimate the mitochondrial respiration rate in the CDK9 (0.5 μM AT7519), OGT (40 μM OSMI-2), and CDK9 + OGT inhibitor-treated cells (24 h). To measure the OCR, inhibitors of the electron transport chain are serially injected to deplete a specific mitochondrial activity. Boxplot presentation of 4 biological replicates. Paired samples Student’s *t*-test was used to assess the statistical significance. C) Heatmap of acyl carnitine levels in response to the indicated treatments (doses and treatment time as in A). Data presented are average of 5 biological replicates. D) Quantitation of acyl carnitines presented in C. The abundance of each acyl carnitine is presented relative to the control sample, which was set to value of 1.

To summarize our findings so far, OGT inhibition affected metabolic processes, while CDK9 inhibition affected transcription. As a single agent, OGT inhibition did not alter the transcriptional program; the major changes that we observed in metabolism were therefore unexpected. In addition, a combination of the 2 inhibitors further suppressed transcription and caused more severe effects on metabolites and the proteome than any single-agent treatment. This suggested that applying a biologically agnostic approach could identify the critical processes affected by co-targeting OGT and CDK9.

### 2.2. Correlation-based network analysis of the transcriptome profiling data

To identify the critical processes affected by co-targeting OGT and CDK9, we chose to use WGCNA as an unbiased systems biology approach. WGCNA calculates weighted correlations and constructs a co-expression gene network from the transcriptome data (Langfelder and Horvath 2008). We used differentially expressed genes from the data presented in Fig. 1D to construct the co-expression network (Fig. 2A). Genes clustered in the brown module positively correlated with control and OGT inhibitor treatment and negatively correlated with CDK9 inhibitor treatment. Essentially, genes that are co-expressed in control samples and OGT inhibitor-treated samples lose this co-expression when either CDK9 alone or, even more so, when both OGT and CDK9 are inhibited. Effects of all of the treatments were most consistent in brown module, which is why we focused on it.

Pathway enrichment analysis of the genes clustered in the brown module revealed a significant enrichment for antigen processing and fatty acid beta-oxidation (Fig. 2B). We mapped these genes to the transcriptomics data and noted that their expression changed robustly in response to CDK9 inhibitor treatment (Fig. 2C). Depletion of OGT activity did not alter this response.

Based on the WGCNA, CDK9 inhibition caused significant effects on transcription, and genes encoding proteins involved in fatty acid oxidation and antigen processing were particularly affected. Depletion of OGT activity together with CDK9 inhibitor did not reverse the effects on transcription of these genes. Earlier, we noted that OGT inhibition preferentially affects metabolism. Overall, we hypothesize that the transcriptional effects caused by CDK9 inhibition divert cells to utilize alternative metabolic resources, which are not available when OGT activity is depleted. If this was the case, then mitochondrial respiration should be reduced by inhibiting OGT and CDK9 combinatorially.

### 2.3. Co-targeting of CDK9 and OGT blocks mitochondrial ATP production

To measure the changes in mitochondrial activity of the CDK9 and OGT inhibitor-treated cells, we used the Seahorse system (Fig. 3A). The Seahorse metabolic flux assay measures the oxygen consumption rate (OCR) by the mitochondrial electron transport chain. In this, inhibitors of the electron transport chain are serially injected to reveal the key parameters of the mitochondrial function. Oligomycin is an ATP synthase inhibitor, carbonyl cyanide-4 (trifluoromethoxy) phenylhydrazone (FCCP) uncouples ATP synthesis from oxygen consumption, and, finally, rotenone and antimycin A target complexes I and III, respectively. This approach allowed us to deduce the impact of inhibiting CDK9 and OGT on mitochondrial respiration.

Cells treated with the CDK9 inhibitor showed reduced ATP-production capacity, and these effects were significantly enhanced by targeting both CDK9 and OGT (Fig. 3B). We note that co-targeting of CDK9 and OGT decreased basal OCR and also reduced maximal respiration and proton leak. However, the coupling efficiency was similar in all samples, which indicated that the mitochondria were not damaged. The data generated using the Seahorse system showed that the ability of prostate cancer cells to generate ATP was severely compromised when both CDK9 and OGT were inhibited.

Our WGCNA analysis identified fatty acid oxidation as the most significantly affected metabolic process, and we therefore evaluated the levels of lipids in OGT and CDK9 inhibitor-treated cells. Lipids can be degraded in the mitochondria to support ATP synthesis, and this process requires carnitine as a carrier of lipids from cytosol to mitochondria (Houten and Wanders 2010). Interestingly, the CDK9 inhibitor increased the amount of acyl carnitines and this effect was antagonized when cells were treated simultaneously with CDK9 and OGT inhibitors (Fig. 3C and D). OGT inhibition alone lowered the abundance of acyl carnitines, which was associated with a modest decline in the OCR.

Together, these data show that mitochondrial activity is decreased when both OGT and CDK9 are inhibited. CDK9 inhibition alters the metabolic activity of cells, and it may be possible to supplement cells with certain metabolites to enhance the efficacy of compounds targeting CDK9.

### 2.4. Pantothenic acid enhances the antiproliferative effects of CDK9 inhibition

**Fig. 4:**
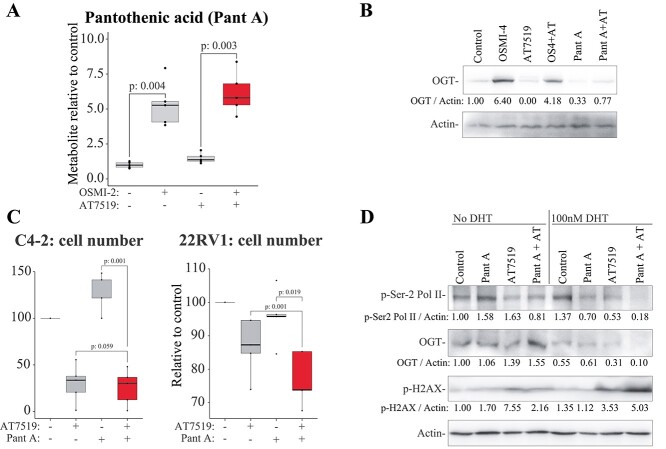
Pantothenic acid and OGT are antagonistic towards each other. A) Pantothenic acid levels are elevated in response to OGT inhibition and OGT + CDK9 inhibition. Data presented are an average of 5 biological replicates. Paired samples Student’s *t*-test was used to assess the statistical significance. B) C4-2 cells were treated with 20 μM OGT inhibitor OSMI-4 or 50 μM pantothenic acid either in the presence or absence of 0.5 μM CDK9 inhibitor AT7519 for 24 h. OGT levels were assessed using western blot and densitometry was used to measure the signal intensity. C) C4-2 (left) and 22RV1 (right) cells were treated with 50 μM pantothenic acid either in the presence or absence of 0.5 μM AT7519 for 4 days and the cell number was assessed using the crystal violet staining. Data presented are an average of 4 biological replicates and paired samples Student’s *t*-test was used to evaluate the statistical significance. D) C4-2 cells were depleted for androgens for 2 days. After this, cells were treated for 24 h as indicated (doses as in B) and analyzed using western blot.

We reanalyzed our metabolite profiling data to identify the metabolites that were strongly affected by OGT and OGT + CDK9 inhibition. The most striking metabolite affected by the OGT inhibitor was pantothenic acid, which was increased 5-fold when OGT activity was depleted, and more than 6-fold for the OGT + CDK9 inhibitor treated cells (Fig. 4A). This contrasted with the impact of combination treatment on acyl carnitines, which restored the levels of these metabolites. Pantothenic acid can be used in a variety of pathways by the cell, including fatty acid synthesis, protein acetylation, steroid synthesis, ketone body biosynthesis, and ATP generation via the citric acid cycle (Tahiliani and Beinlich 1991; Pietrocola et al. 2015). mRNAs belonging to these processes were significantly altered in response to CDK9 inhibitor treatment, but we did not identify a particular process that could be antagonized by OGT inhibition (Supplementary Fig. 4). We used pantothenic acid in combination with CDK9 inhibitor to establish if this combinatorial strategy induces antiproliferative effects on prostate cancer cells. Compounds targeting CDK9 antagonize OGT inhibitor-induced upregulation of OGT, which in part explains the combinatorial toxicity between CDK9 and OGT inhibitors (Itkonen et al. 2020; Hu et al. 2021). We confirmed these effects and noted that treating C4-2 cells with pantothenic acid alone and pantothenic acid + AT7519 decreased the expression of OGT (Fig. 4B). Combining pantothenic acid with CDK9 inhibitor significantly, albeit modestly, decreased the proliferation of prostate cancer cells (5%–10% decline, Fig. 4C). As a single agent, pantothenic acid increased the cell number of the C4-2 cells. Interestingly, pantothenic acid dose-dependently increased the number of the C4-2 cells maintained in the normal serum but not in the absence of androgens (Supplementary Fig. 5). The presence and absence of androgens appears to be important for the effects of pantothenic acid on prostate cancer cells. Therefore, we cultured cells under conditions of androgen deprivation for 2 days prior to treating them with dihydrotestosterone to activate the AR. We then evaluated the effects of the CDK9 inhibitor and pantothenic acid on protein markers of interest. Previously, we have shown that CDK9 inhibition decreases RNA Pol II phosphorylation and causes DNA damage, both of which are enhanced when OGT activity is simultaneously depleted (Itkonen et al. 2020; Gondane et al. 2022). Combination of pantothenic acid with AT7519 elicited a further decrease in RNA Pol II phosphorylation and an increase in the DNA damage marker p-H2AX only in the presence of androgens (Fig. 4D).

In conclusion, we have shown that OGT is required for the pro-survival metabolic rewiring of prostate cancer cells treated with CDK9 inhibitor. Pantothenic acid (vitamin B5) is a water-soluble vitamin, which can enhance the efficacy of CDK9 inhibition.

## 3. Discussion

In this study, we have used a multiomics approach to demonstrate that OGT is required for metabolic homeostasis in both basal conditions and in response to transcriptional stress caused by CDK9 inhibition. The starting point for our study was the combinatorial lethality screen, which discovered that co-targeting of OGT and CDK9 is toxic to prostate cancer cells (Itkonen et al. 2020); our goal in this project was to understand why this is. The combinatorial lethality screen was performed in an androgen-dependent LNCaP cell line (Itkonen et al. 2020), which is why we selected this model for the multiomics profiling reported here. Androgen-dependent prostate cancers can be successfully treated with anti-androgens; however, a significant number of patients develop castration-resistant prostate cancer (CRPC) (Sathianathen et al. 2018; Sandhu et al. 2021). Currently, there are no curative treatment-strategies against the CRPC, which is why we selected models of CRPC for the validation experiments in our study. Based on the data presented here, and in previous studies (Itkonen et al. 2020; Hu et al. 2021; Gondane et al. 2022), CDK9 inhibition triggers adaptive responses at multiple levels to allow cancer cell survival. OGT appears important to maintain RNA Pol II activity, because co-targeting of OGT and CDK9 further suppresses global transcription (Fig. 1D). As an acute response, CDK9 inhibition causes DNA damage, and OGT is required to resolve this (Gondane et al. 2022). In addition, a small number of mRNAs encoding the splicing machinery are increased when CDK9 is inhibited, and these mRNAs depend on OGT (Hu et al. 2021). Here, we discovered that OGT is also required for the metabolic adaptation when CDK9 is inhibited (Fig. 3). Clearly, OGT acts at multiple points to enable cancer cells’ adaptive response to CDK9 inhibition. Dissecting these responses has the potential to identify those that are cancer cell-selective, which can subsequently be targeted in combinatorial treatment strategies with CDK9 inhibitors.

OGT inhibition reduces the levels of most metabolites, and the samples treated with the OGT inhibitor form a distinct cluster based on the PCA (Fig. 1A and C). In contrast, CDK9 inhibitor does not change the gross metabolic program from the basal or OGT inhibitor treated, as determined using the PCA (Fig. 1A). Both the PCA analysis and more detailed mapping of the metabolic profiles show that the metabolic dynamics are governed by OGT in prostate cancer cells.

Our experiments show that the effects of CDK9 inhibition extend beyond an impact on transcription. CDK9 is the kinase that promotes productive transcription elongation (Chou et al. 2020); accordingly, we find that depletion of CDK9 activity downregulates the vast majority of mRNAs (Fig. 1D). In addition to transcription, depletion of CDK9 activity has been shown to affect metabolism in prostate cancer cells (Itkonen, Poulose, et al. 2019a). Here, we show that the CDK9 inhibitor-induced increase in acyl carnitines is antagonized by inhibiting OGT (Fig. 3C and D). CDK9 inhibitor-induced effects on metabolism reflect, at least in part, a decrease in glucose uptake and the subsequent decline in the ATP levels (Huang et al. 2021). In addition, CDK9 inhibition has been shown to decrease the production of the reactive oxygen species (ROS), which may be due to decreased ATP production. We hypothesize that in response to the decline in CDK9 activity, cancer cells decrease glucose uptake and ATP synthesis to suppress production of ROS and thereby limit the DNA damage. This hypothesis is supported by the fact that CDK9 inhibition causes DNA damage and sensitizes cancer cells to genotoxic agents (Storch and Cordes 2016; Nepomuceno et al. 2017; Song et al. 2019; Gondane et al. 2022).

Increased DNA damage attracts the immune system to the affected cells. In this study, we provide evidence at both the transcriptional and protein levels that the inhibition of CDK9 activity can boost the immunogenicity of cancer cells. Interestingly, using an unbiased systems biology approach, we discovered that “antigen processing and presentation of exogenous peptide” is the most enriched pathway in response to targeting CDK9 (Fig. 2B). In addition, we noted that co-targeting of OGT and CDK9 stabilizes the IRF1 transcription factor (Supplementary Fig. 3B). IRF1 has the potential to activate both the adaptive and innate immune responses (Ivashkiv and Donlin 2014). In the future, it will be important to further explore this interplay using more complex model systems encompassing immunological responses such as syngeneic mouse models.

We found that pantothenic acid (vitamin B5) is the metabolite that is most increased when both OGT and CDK9 are inhibited (Fig. 4A). Vitamin B5 has been investigated in clinical trials due to its potential to regulate the immune system (Axelrod 1981; Gheita et al. 2020; Peterson et al. 2020). In our experiments, we noted that co-treatment of prostate cancer cells with vitamin B5 and CDK9 inhibitor induces combinatorial antiproliferative effects and further increased DNA damage, which were associated with decreased OGT expression (Fig. 4). In the future, it may be possible to use vitamin B5 as an adjuvant to boost the antitumor effects of DNA damaging agents, including CDK9 inhibitors.

## 4. Materials and methods

### 4.1. Cell culture, compounds, proliferation assays, preparation of cell lysates, and isolation of RNA

LNCaP, 22RV1, and C4-2 cell lines were obtained from the American Tissue Culture Collection. Cells were maintained in 10% fetal bovine serum (FBS) in RPMI medium. RNA isolation was performed using the illustraMiniSpin-kit (GE Healthcare) according to manufacturer’s instructions, and cDNA was synthesized using the qScript cDNA Synthesis Kit (Quantabio). For androgen-starvation experiments, cells were maintained in phenol red-free RPMI medium supplemented with charcoal-stripped FBS. AT7519 was purchased from Selleckchem for reverse-phase protein array and metabolite profiling experiments, and for the other experiments, from MedChemExpress. OSMI-2 was synthesized in Professor Suzanne Walker’s laboratory (Harvard Medical School), while OSMI-4 (Martin et al. 2018) and dihydrotestosterone were purchased from MedChemExpress. Cell lysates for western blotting were prepared as previously described (Itkonen and Mills 2013), and antibodies used are from Cell Signaling technology: p-Ser2-RNA Pol II (13499) and OGT (24083); from Santa Cruz Biotechnology: p-H2AX (517348); and from Abcam: RL2 (ab2739) and Actin (ab49900). Signal intensity of western blot (densitometry) was determined using Image Lab version 6.0 (Bio-Rad). Cell number was assessed using crystal violet staining assay as previously described (Barkovskaya et al. 2020).

### 4.2. Metabolite profiling and reverse-phase protein arrays

For metabolic profiling, LNCaP cells were treated for 24 h with 0.5 μM AT7519, 40 μM OSMI-2, or combination of both. Cells were harvested by washing with PBS, trypsinization, and centrifuged at 4,000 rpm for 5 min at 4°C. The pellet was washed with PBS, centrifuged again 4,000 rpm for 5 min at 4°C, and, finally, the pellet was washed with water. The cells were stored at −80°C until handed over to the metabolite profiling facility. Targeted metabolite profiling was purchased as a service from FIMM Metabolomics/Lipidomics/Fluxomics Unit (Helsinki, Finland). For reverse-phase protein array profiling, the cells were treated for 24 h with 0.5 μM AT7519, 40 μM OSMI-2, or combination of both, and the samples were prepared as previously described (Itkonen, Poulose, et al. 2019a). Reverse-phase protein array profiling was purchased as a service from MD Anderson.

### 4.3. Multiomics profiling, data preprocessing, and analysis

The RNA-seq data for LNCaP cells treated for 4 h with inhibitors of CDK9, OGT, or the combination of both compounds were downloaded from GEO database (GSE116778) (Itkonen et al. 2020). We used the deposited normalized count matrix in our analysis. Genes with *P*-value less than 0.05 were considered significant and used for constructing the co-expression network using WGCNA (explained in the section below). Gene lists for the various acetyl-CoA-related pathways were downloaded from Gene Set enrichment Analysis database (Subramanian et al. 2005). All the volcano and bar plots were made in R Studio v4.1.1.

### 4.4. WGCNA and module identification

We constructed weighted gene co-expression networks of previously generated RNA-seq data (Itkonen et al. 2020) using WGCNA package in R (Langfelder and Horvath 2008). The normalized read counts matrix was used to identify the outliers from the samples. As no outliers were identified from the preprocessed data, all the samples were used for network construction. Next, a power of β = 7 was selected as the soft threshold to ensure a scale-free network. Pearson correlation coefficient was used to evaluate weighted co-expression relationships among the genes. The identified significant modules were subjected to pathway enrichment analysis in Enrichr (KEGG pathways) (Kuleshov et al. 2016).

### 4.5. Seahorse metabolic flux analysis

Seahorse XFe 96 instrument was used to measure the oxygen consumption rate (OCR). Equal number of cells were plated, and the next day, the cells were treated for 24 h with 0.5 μM AT7519, 40 μM OSMI-2, or combination of both. Prior to the assay, Cartridge of the instrument was equilibrated overnight and loaded with Oligomycin, FCCP, and rotenone/antimycin A in 2 μM, 1 μM, and 0.5 μM concentration, respectively. Results were analyzed using the Seahorse Wave software.

## Supplementary Material

Supplementary_figures_and_supplementary_figure_legends_cwac038
