# Supplementary material for: O-GlcNAc transferase maintains metabolic homeostasis in response to CDK9 inhibition
Source: Glycobiology. 2022 Jun 16;32(9):751–9. doi: 10.1093/glycob/cwac038 (PMC9387508; doi:10.1093/glycob/cwac038)
Supplement: Supplementary_figures_and_supplementary_figure_legends_cwac038 [file supplementary_figures_and_supplementary_figure_legends_cwac038.pdf]

Supplementary figures and supplementary figure legends

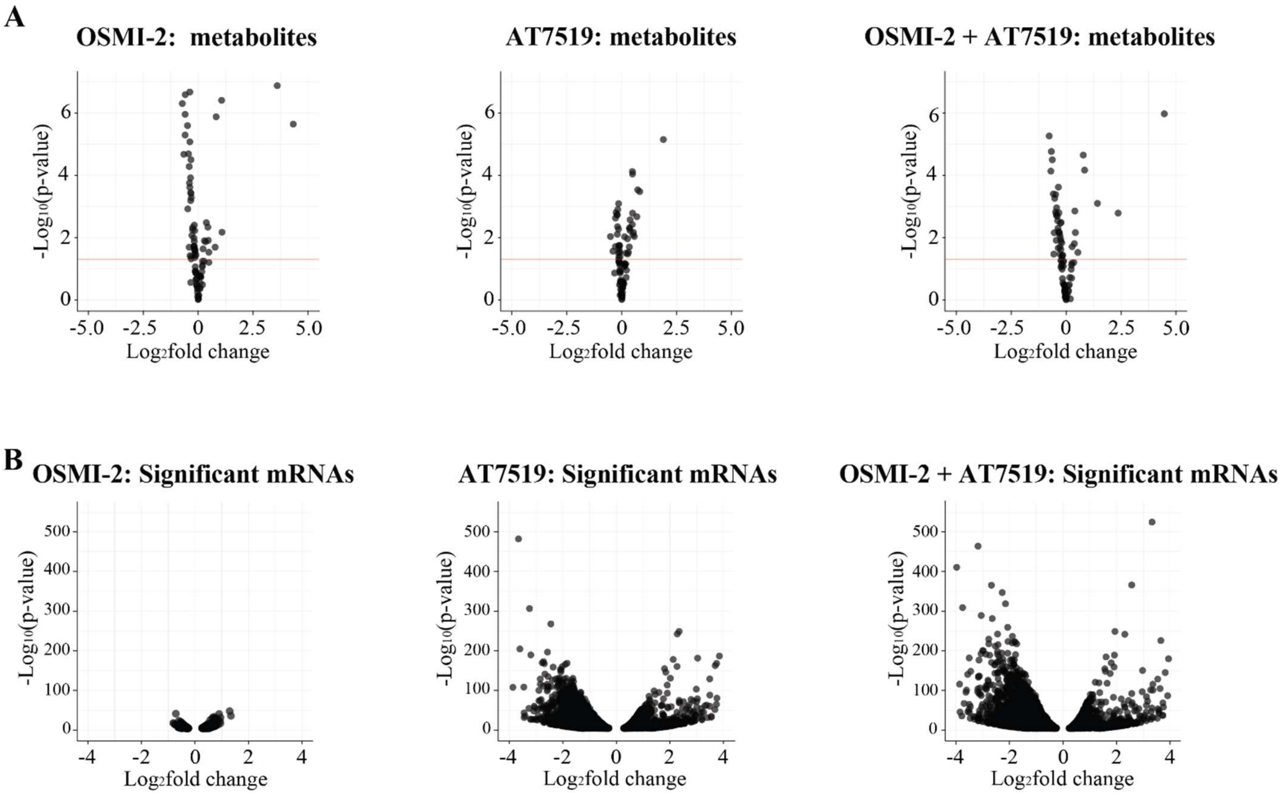

Supplementary figure 1. Individual volcano plots of the data presented in the main figure 1. OGT inhibition affects metabolic processes in prostate cancer cells. **A)** LNCaP cells were treated for 24 hours with inhibitors of CDK9 (0.5µM AT7519), OGT (40µM OSMI-2) and CDK9+OGT followed by metabolite profiling. **B)** Fold changes in gene expression in the AT7519, OSMI-2 and combination treatments. Doses as in A.

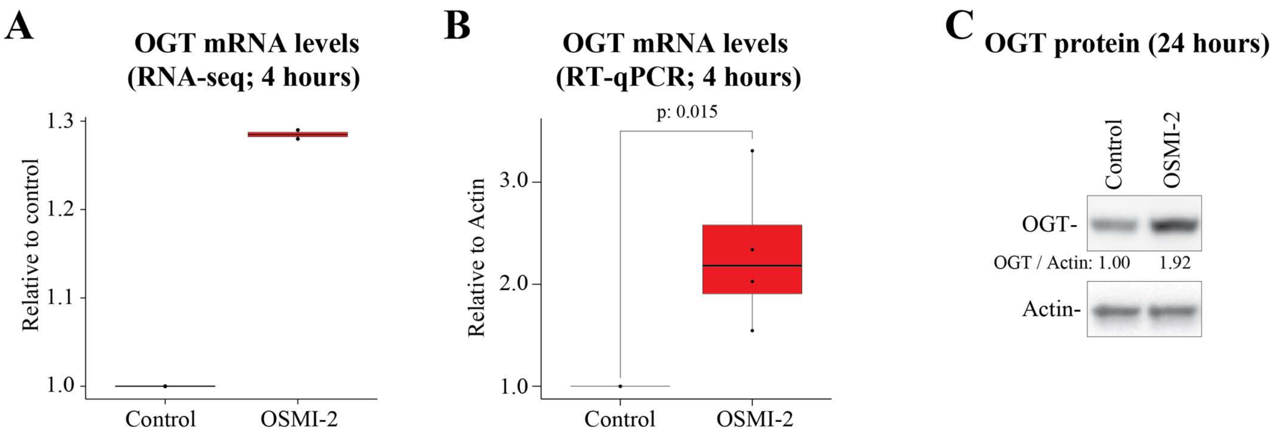

Supplementary figure 2. OGT inhibition using OSMI-2 induces OGT at the mRNA and protein levels. A) LNCaP cells were treated with 40µM OSMI-2 for 4 hours and analyzed using RNA-seq. Raw read counts of OGT gene expression values normalized to control (DMSO) treated samples is presented (p-value: 2.56E-07). **B)** LNCaP cells were treated with 40µM OSMI-2 for 4 hours, mRNA isolated and analyzed using RT-qPCR. Data shown represents four biological replicates. Student's paired sample, two-tailed t-test was used to evaluate the significance. **C)** LNCaP cells were treated with 40µM OSMI-2 for 24 hours and analyzed using western blot. Densitometry was used to quantitate the signal intensity.

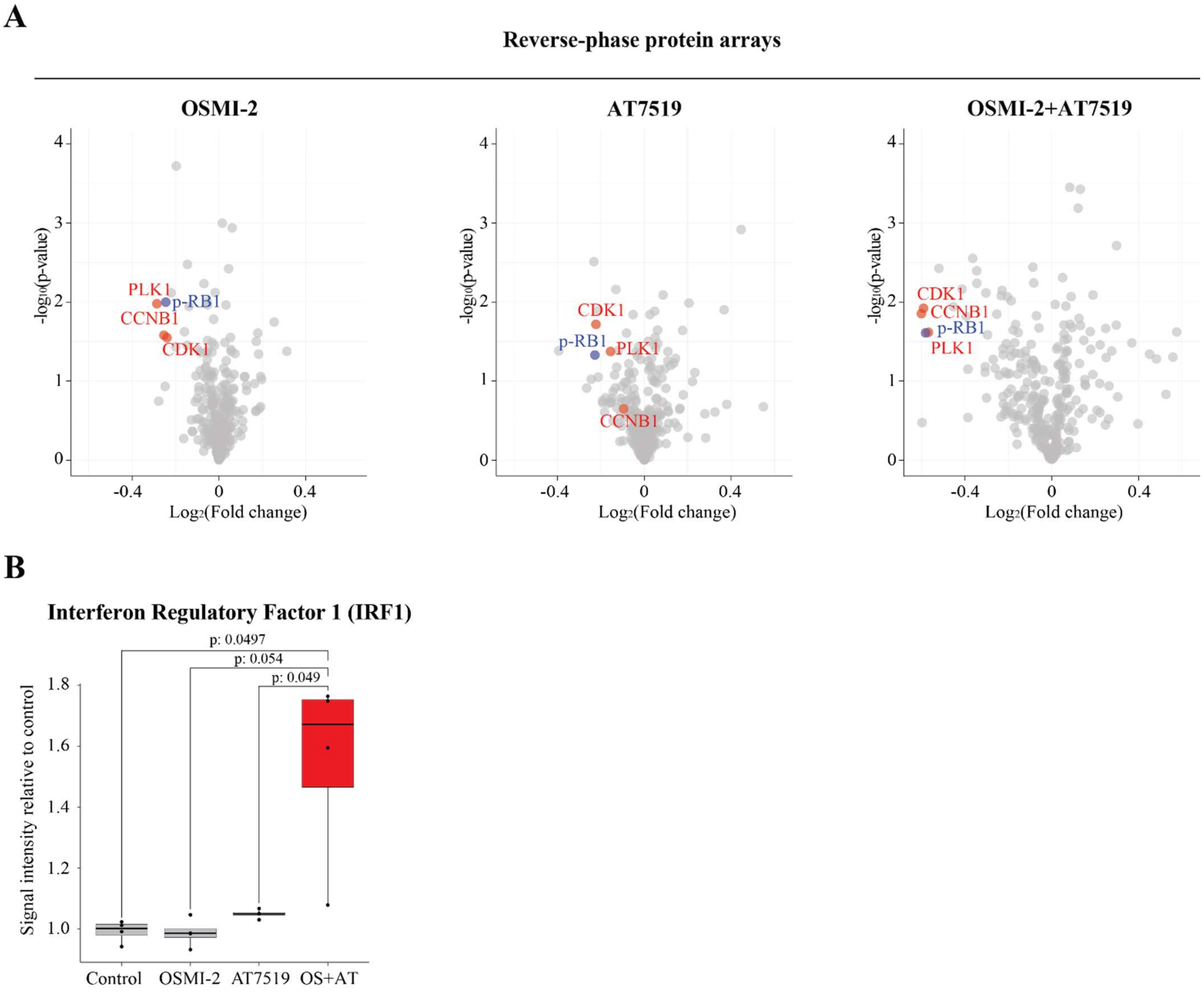

Supplementary figure 3. Reverse-phase protein array (RPPA) profiling of LNCaP cell response to OGT and CDK9 inhibition. A) LNCaP cells were treated for 24 hours with inhibitors of CDK9 (0.5µM AT7519), OGT (40µM OSMI-2) and CDK9+OGT, after which samples were analyzed using RPPA for 302 antibodies. Highlighted are the proteins known to be affected by OGT inhibition (red; CDK1, cyclin B1 and PLK1) and CDK9 inhibition (blue; phosphorylation of RB1. Data shown is an average of four biological replicates. **B)** Signal for IRF1 antibody after the indicated treatments. Data shown is an average of four biological replicates and paired samples Student's t-test was used to assess the statistical significance.

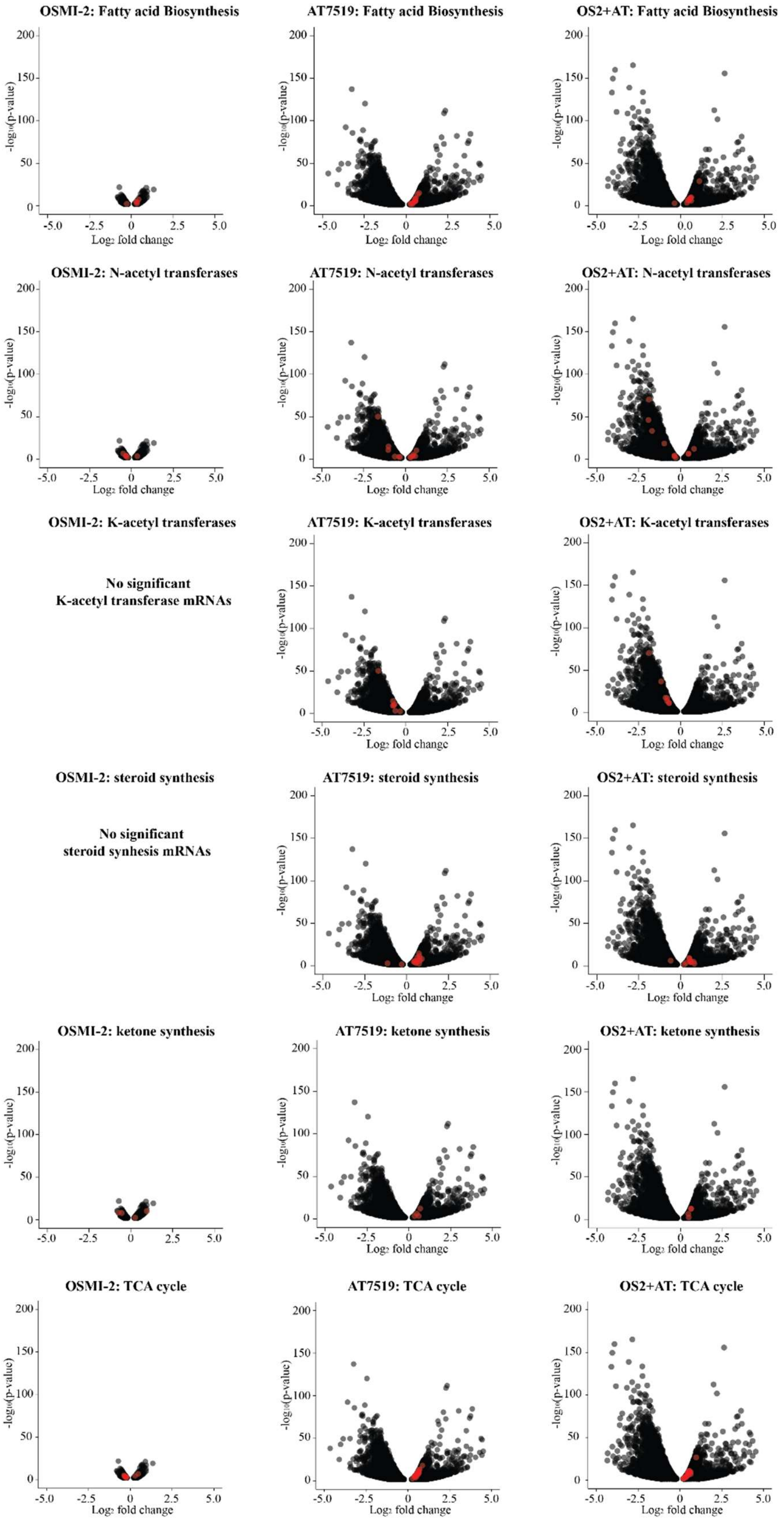

Supplementary figure 4. OGT and CDK9 inhibitor effects on genes related to acetyl-CoA metabolism. Gene lists for the various acetyl-CoA related pathways were downloaded from Gene Set enrichment Analysis database (Subramanian, A., Tamayo, P., et al. 2005). All significant genes are presented in black color, while the genes belonging to the particular pathway are highlighted in red.

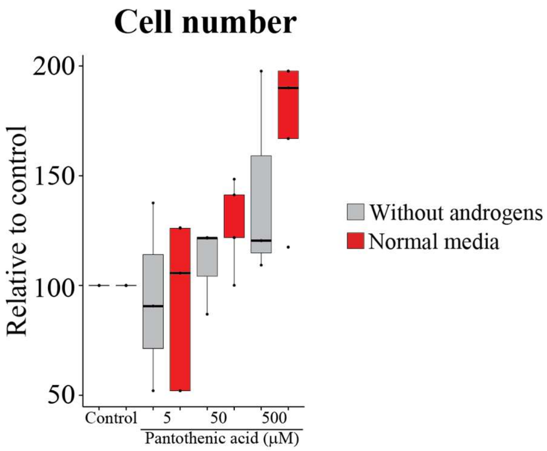

Supplementary figure 5. Pantothenic acid promotes proliferation of prostate cancer cells. C4-2 cells were treated as indicated for four days. Data shown is an average of four biological replicates.

References

Subramanian A, Tamayo P, Mootha VK, Mukherjee S, Ebert BL, Gillette MA, Paulovich A, Pomeroy SL, Golub TR, Lander ES*, et al.* 2005. Gene set enrichment analysis: a knowledge-based approach for interpreting genome-wide expression profiles. *Proc Natl Acad Sci U S A*, 102:15545-15550.
